# Paeoniflorin Inhibits LPS-Induced Activation of Splenic CD4^+^ T Lymphocytes and Relieves Pathological Symptoms in MRL/lpr Mice by Suppressing IRAK1 Signaling

**DOI:** 10.1155/2022/5161890

**Published:** 2022-11-23

**Authors:** Lina Ji, Shenglong Wang, Shan Wu, Jie Bao, Guanqun Xie, Yan Zhang, Liping Xu, Na Lin, Jian Wang, Yongsheng Fan, Danqing Fu, Qiaoding Dai

**Affiliations:** ^1^The First Affiliated Hospital of Zhejiang Chinese Medical University, Hangzhou 310003, Zhejiang Province, China; ^2^The First School of Clinical Medicine, Zhejiang Chinese Medical University, Hangzhou 310053, Zhejiang Province, China; ^3^The First Affiliated Hospital of Hangzhou Normal University, Hangzhou 310015, Zhejiang Province, China; ^4^School of Basic Medical Sciences, Zhejiang Chinese Medical University, Hangzhou 310053, Zhejiang Province, China

## Abstract

Interleukin-1receptor-associated kinase 1 (IRAK1) plays a critical role in systemic lupus erythematosus (SLE). It was reported that SLE was associated with an inflammatory response mediated by defective immune tolerance, including overproduction of autoantibodies, chronic inflammation, and organ damage. Previous reports stated paeoniflorin (PF) had an immunosuppressive effect. The purpose of this study was to determine the anti-inflammatory effect of PF in SLE and its underlying mechanisms. Followed by induced with lipopolysaccharide (LPS), the splenocytes and the isolated CD4^+^ T lymphocytes of MRL/lpr mice were divided into three groups: control group, LPS group, and LPS + PF group, respectively. MRL/MP mice were used as the control group (treated with distilled water). The MRL/lpr mice were randomly divided into three groups: the model group (treated with distilled water), the prednisone group, and the PF group. The MRL/lpr mice were treated with prednisone acetate (5 mg/kg) and PF (25, 50, and 75 mg/kg) for eight weeks. Subsequently, ELISA, qRT-PCR, western blotting, HE, and Masson staining were performed to detect various indicators. The results of Cell Counting Kit-8 (CCK-8) showed that 10 *μ*g/mL of LPS had the optimum effect on cell viability, and 50 *μ*mol/L of PF had no obvious cytotoxicity to LPS-treated cells. PF reduced the expression level of IRAK1-nuclearfactor-*κ*B (NF-*κ*B) and its downstream inflammatory cytokines in the splenocytes and CD4^+^ T lymphocytes of MRL/lpr mice stimulated by LPS, especially in the latter. The serum antibody contents in the PF group mice were reduced, and the kidney damage was also alleviated accordingly. Moreover, the IRAK1/inhibitor of the nuclear factor-*κ*B kinase (IKK)/NF-*κ*B inhibitor (I*κ*B)/NF-*κ*B pathways was found to be involved in the anti-inflammation effect of PF in the kidney and spleen. In conclusion, it is thought that PF may have the potential to be used as a therapeutic agent to reduce the inflammatory activity of SLE. Inhibition of the IRAK1-NF-*κ*B pathway may help formulate novel therapeutic tactics for SLE.

## 1. Introduction

Systemic lupus erythematosus (SLE) is a chronic autoimmune disease that affects multiple organ systems. It was reported that SLE was associated with an immune tolerance deficiency-mediated inflammatory response, including overproduction of autoantibodies, chronic inflammation, and organ damage [1,2]. Glucocorticoids and immunosuppressive agents are effective in the treatment of SLE. However, there are many serious side effects, such as metabolic disorders, susceptibility to infection, and withdrawal reactions [3]. Biotherapies, such as belimumab, limit their extensive clinical application due to antisingle subtype and expensive price [4]. Recently, increasing attention has focused on revealing SLE pathogenesis, especially its role in inflammatory signals.

Interleukin-1receptor-associated kinase 1 (IRAK1) is a serine/threonine kinase that targets the downstream of the interleukin-1 receptor (IL-1R) and participates in the activation of the autoimmune Toll-like receptor (TLR) signaling pathway [5,6]. TLRs have been identified as therapeutic targets for SLE, among which TLR4 is found to contribute to the auto-inflammatory diseases [7,8]. IRAK1 is a crucial signal molecule involved in the TLR4 signal transduction pathway [3]. The related connexin IRAK1 will be activated by TLR through the myeloid differentiation primary response 88 (MyD88)-dependent signaling pathway, which leads to the activation of tumor necrosis factor (TNF) receptor-associated factor 6 (TRAF6) and inhibitor of nuclear factor-*κ*B (NF-*κ*B) kinase (IKK) [9]. Eventually, NF-*κ*B is activated to induce the synthesis and secretion of inflammatory cytokines [10,11]. It was reported by multiple studies that the IRAK1 gene polymorphism was related to the susceptibility of human SLE, which might be a potential therapeutic target for SLE [12–14]. However, the exact functional outcome of the IRAK1 gene polymorphism on SLE susceptibility still needs further research.

It was found that the misfunction of the NF-*κ*B pathway was engaged in autoimmune diseases and inflammatory diseases [15,16]. NF-*κ*B was reported to be involved in the pathogenesis of SLE, in which IRAK1 played a crucial role in the abnormal activation of NF-*κ*B in inflammatory diseases [9]. Consequently, inhibiting the activity of IRAK1-NF-*κ*B may be an effective strategy for developing new therapies for SLE or other inflammatory diseases. However, there are few studies on the function of the IRAK1-NF-*κ*B signal in SLE.

At present, traditional Chinese medicine (TCM) for the clinical treatment of autoimmune inflammatory diseases has shown excellent development potential [17]. The Jieduquyuziyin Prescription (JP), containing ten traditional Chinese herbs, is clinically used to treat SLE. Our previous research confirmed that JP could inhibit inflammation, alleviate the side effects of clinical drugs, and reduce the incidence of infection [18]. Recent clinical studies have found that JP could reduce the side effects and enhance the efficacy of glucocorticoids (GC) in the treatment of SLE [19,20]. Importantly, paeoniflorin (PF) is the main active ingredient of JP [21]. PF was the chief biologically active ingredient in the herb *Paeonia lactiflora Pall* (Chinese peony), which had good anti-inflammatory and immune-regulating functions [22,23]. PF could regulate the activation and proliferation of T lymphocytes and participate in the inflammation and immune process of autoimmune diseases through signal pathways such as the NF-*κ*B pathway [24]. Published studies state that PF might prevent the depressive behavior caused by SLE through the high mobility group protein B1 (HMGB1)/TLR4/NF-*κ*B pathway [25]. Due to the multiple roles of PF in anti-inflammatory and immune regulation, it is speculated that PF may be an efficacious drug for treating SLE. Nevertheless, the definite contribution of PF to the pathogenesis of SLE inflammation has not been fully elucidated.

Previously, we only studied the related pathways of IRAK1-NF-*κ*B in macrophages of MRL/lpr mice at the cellular level [26]. Studies have reported that IRAK1 inhibitors can inhibit inflammatory signals in splenic monocytes of lupus mice [27]. However, questions like whether IRAK1 inhibitors could function on CD4^+^ T lymphocytes and whether PF has the same effect as IRAK1 inhibitors have not been answered yet. Thus, our study aimed to explore the function of PF on infection during the pathogenesis of SLE in vivo and in vitro. Assuming that inhibition of IRAK1 activity might relieve inflammation in lupus mouse models, we studied the lipopolysaccharide (LPS)-induced splenocytes, CD4^+^ T lymphocytes, and pathological symptoms of MRL/lpr mice. Overall, we explored the anti-inflammatory effect of PF on MRL/lpr mice through the IRAK1-NF-*κ*B signaling pathway.

## 2. Materials and Methods

### 2.1. Reagents and Chemicals

LPS was purchased from Sigma-Aldrich (St. Louis, MO). PF was purchased from Yuanye (S31585, HPLC ≥98%, Shanghai, China). Antibodies against IRAK1 (ab238, polyclonal antibody), p-IRAK1 (ab218130, polyclonal antibody), IKK*α*/*β* (ab178870, monoclonal antibody), I*κ*B*α* (ab32518, monoclonal antibody), p-I*κ*B*α* (ab133462, monoclonal antibody), NF-*κ*B (ab32536, monoclonal antibody), and p-NF-*κ*B (ab86299, polyclonal antibody) were purchased from Abcam (Cambridge, UK). Antibodies against p-IKK*α*/*β* (2697, monoclonal antibody), *α*-tubulin (2144, polyclonal antibody), and anti-mouse/rabbit IgG (7076/7074, HRP-linked Antibody) were purchased from Cell Signaling Technology (Beverly, MA, USA). A chemiluminescent horseradish peroxidase (HRP) substrate was purchased from Millipore (Burlington, MA, USA).

### 2.2. Isolation and Culture of Splenocytes and CD4+ T Lymphocytes

The spleens were obtained from MRL/MP and MRL/lpr mice and placed in RPMI1640 (Gibco, CA, USA) supplemented with penicillin (100 unit/mL) and streptomycin (100 *μ*g). A single cell suspension of spleen in each group was prepared with a cell strainer (40 *μ*m, Corning, NYC, USA). After centrifugation (1200 rpm, 5 min, 4°C), the supernatant was removed, and cell lysis buffer (Beyotime, Shanghai, China) was added. Next, cells were washed twice in RPMI1640 containing 10% fetal bovine serum (FBS, Gibco, CA, USA) and splenocytes were obtained after centrifugation [28]. Finally, splenocytes are a suspension of single cells from the spleen [29].

The collected splenocytes were resuspended in a buffer (Miltenyi Biotec, Bergisch Gladbach, Germany) according to the operating instructions of the CD4^+^ T cell isolation kit (Miltenyi Biotec, Bergisch Gladbach, Germany). The cells were incubated with Biotin-Antibody Cocktail and Antibiotin MicroBeads successively. Ultimately, the unlabeled cells collected by the LS Column (Miltenyi Biotec, Bergisch Gladbach, Germany) were CD4^+^ T cells.

### 2.3. Flow Cytometry

The collected CD4^+^ T cells were resuspended in 1640 medium containing 10% FBS and centrifuged to discard the supernatant. Then, anti-mouse CD4 and PE and anti-mouse CD3 and FITC (Multi Sciences, Hangzhou, China) were added after the cells were washed with phosphate-buffered saline (PBS). Then, the cells were mixed slowly and incubated in the dark. Finally, they were detected by a flow cytometer (BD, CA, USA).

### 2.4. Cell Viability Analysis

Splenocytes and CD4^+^ T lymphocytes were cultured alone or with PF (10, 25, 50 *μ*mol/L) for 24 h after induction with LPS (10 *μ*g/mL). Cell viability was detected by counting kit-8 (CCK-8, Beyotime, Shanghai, China). Cells treated with vehicle (1640 supplemented with 10% FBS) were taken as the control group. The absorbance at 562 nm was detected in a microplate reader (PerkinElmer, EnSpire, MA, USA).

### 2.5. Mice and Treatments

MRL/lpr and MRL/MP mice (6-7 weeks old, female, 20–30 g) were purchased from SLAC Laboratory Animal Co., Ltd. (Shanghai, China) and raised in the animal research center of Zhejiang Chinese Medical University under specific pathogen-free (SPF) conditions at a constant temperature of 25°C in a 12-h light/dark cycle with a humidity of 40–60%.

All mice were randomly divided into the following groups: control group, model group, prednisone group (PDN group, a positive control drug), and PF group, and were given drugs and diluted water by intragastric administration for eight weeks as described previously [21]. MRL/MP mice taken as a control group and MRL/lpr mice taken as a model group were fed with distilled water. Prednisone tablets (Xainju Pharma, Zhejiang, China) suspension was prepared with distilled water and gavaged to MRL/lpr mice at a dose of 5 mg/kg for eight weeks [30,31]. The mice in the PF group were given 25, 50, and 75 mg/kg of PF dissolved in distilled water, respectively [25]. In the end, the spleen, kidney, urine, and blood were harvested after all the mice were euthanized with carbon dioxide [18].

All the above animal studies were approved by the Animal Experiment Ethics Committee of Zhejiang Chinese Medical University. The ethics approval/permit number for the use of animals in this study is IACUC-20181119-04/SYXK2018-0012.

### 2.6. Enzyme-Linked Immunosorbent Assay (ELISA)

Cells were grown in 6-well plates and treated with LPS alone or with paeoniflorin. The levels of TNF-*α* and IL-6 in cell supernatant were detected by ELISA kits (NOVUS biologicals, SLLC, USA) according to the manufacturer's instructions.

The supernatant of mouse urine was collected by centrifugation, and the concentration of urine protein was determined according to the urine protein and urinary creatinine test kit (Jiancheng, Nanjing, China). The venous blood of each mouse was kept at room temperature, and the serum was obtained after centrifugation. Afterwards, the concentration of anti-dsDNA and anti-nRNP/Sm in serum was detected according to the instructions of the anti-dsDNA ELISA Kit and anti-nsRNP/Sm ELISA Kit (Euroimmun, Lubeck, Germany).

### 2.7. Kidney Histopathology

The kidney specimens were fixed by using 4% paraformaldehyde and embedded in paraffin wax. Paraffin segments were cut and stained with hematoxylin-eosin (H&E) and Masson staining using standard procedures. Consequently, the digital pathological section scanning system (Hamamatsu Photonics K.K., Shizuoka Pref., Japan), NDP. View 2 Plus (Hamamatsu Photonics K.K., Shizuoka Pref., Japan), and light microscopy (Motic, Xiamen, China) were used to observe and analyze the pathologic change.

### 2.8. RNA Extraction, cDNA Synthesis, and Quantitative Real-Time PCR (Rt-qPCR)

Total RNA was extracted from splenocytes, CD4^+^ T lymphocytes, spleen and kidney tissues by RNAiso Plus (Takara Bio Inc., Otsu, Japan). Then, the RNA was reverse-transcribed into cDNA. TB green premix ex taq (Takara Bio Inc., Otsu, Japan) was used for Rt-qPCR analysis. Rt-qPCR was performed by the Roche LightCycler 96 SW1.1 instrument (Roche, Basel, Switzerland). Ultimately, the relative expression of each target gene was detected by the 2^^(−ΔΔCt)^ method after normalization by glyceraldehyde 3-phosphate dehydrogenase (GAPDH, Sangon Biotech, Shanghai, China) [21]. Sequences of the PCR primers were as follows: IRAK1 (5′-GGTCCCTGTCTCTTCCCTTC-3′ and 5′-GAGGAAGGAATTCAGCCTTTG-3′), NF-*κ*B (5′-GCCGTGGAGTACGACAA-3′ and 5′-CGGTTTCCCATTTAGTATGT-3′), tumor necrosis factor *α* (TNF-*α*) (5′-ACCAGACACCTCAGGGCTAA-3′ and 5′-TGTTGGGGAGAAGGAGAATG-3′),Interleukin-6 (IL-6) (5′-TACCACTCCCAACAGACCTG-3′ and 5′-GGTACTCCAGAAGACCAGAGG-3′),Interleukin-1*β* (IL-1*β*) (5′-TCAGGCAGGCAGTATCACTC-3′ and 5′-AGCTCATATGGGTCCGACAG-3′).

### 2.9. Western Blotting

The total proteins of splenocytes, CD4^+^ T lymphocytes, spleen, and kidney tissues from different groups were extracted with the Qproteome Mammalian Protein Prep Kit (Qiagen, Dusseldorf, Germany). The BCA Protein Assay Kit (Biosharp, Hefei, China) was applied to determine the protein concentrations. The protein membranes were blocked at room temperature, and then incubated with primary antibodies diluted in a blocking solution overnight. Afterwards, the membranes were incubated with IgG antibodies (1 : 1,000) and finally developed the membranes with ECL Substrate (Millipore Corp., Billerica, MA, USA). The signals were quantified by an imager (Proteinsimple, CA, USA), and the ratio of protein band to *α*-tubulin was quantified by FluorChem FC3 software (Proteinsimple, CA, USA).

### 2.10. Statistical Analysis

The data were presented as mean ± standard deviation (SD). One-way analysis of ANOVA and *t*-test were determined by the software GraphPad Prism 6 (GraphPad Software Inc., San Diego, CA, USA) and SPSS 22.0 (IBM SPSS, Chicago, IL, USA). *p* < 0.05 was statistically significant.

## 3. Results

### 3.1. Potential Cytotoxicity and Cytoprotection of PF in LPS-Induced CD4^+^ T Lymphocytes of MRL/lpr Mice

**Figure 1:**
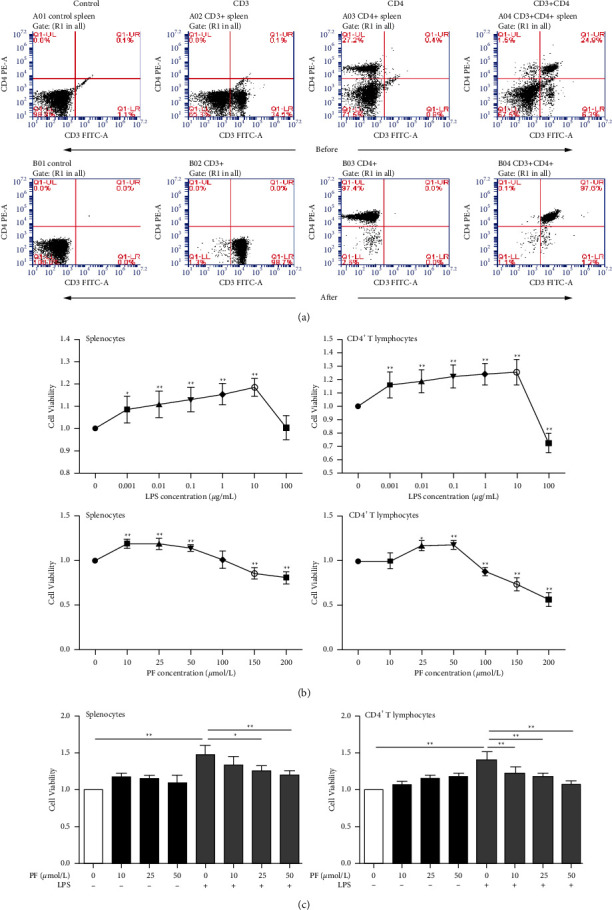
Effect of LPS and PF on the cell viability of splenocytes and spleen CD4^+^ T lymphocytes for 24 h. (a) The purity of CD4^+^ T lymphocytes was identified by flow cytometry and staining with anti-mouse CD3*ε* (FITC) and anti-mouse CD4 (PE). The purity of CD4^+^ T lymphocytes before and after sorting was tested and compared. (b) Splenocytes and spleen CD4^+^ T lymphocytes viability were assessed by CCK-8 assay after treating with LPS or PF for 24 h (*n* = 6). (c) Splenocytes and splenic CD4^+^ T lymphocytes were treated with PF (10, 25, 50 *μ*mol/L) or without LPS (10 *μ*g/mL) for 24 h (*n* = 4). The results were shown as mean ± S.D. ^*∗*^ and ^*∗∗*^ indicate significant differences at the levels of *p* < 0.05 and *p* < 0.01, respectively, compared to untreated cells or solely LPS-treated cells.

It has been reported that lupus-susceptible mice carry a great quantity of activated T cells, and CD4^+^ T cells in SLE have signal defects [32,33]. To further elucidate the mechanism of PF on CD4^+^ T lymphocytes in MRL/lpr mice, we adopted the LPS induction method. Firstly, we purified and identified the extracted CD4^+^ T lymphocytes. As shown in Figure 1(a), the ratio of CD3*ε* before sorting was 34.5%, the ratio of CD4 was 27.2%, and the ratio of CD3*ε*+CD4 was 24.8%. After sorting, the ratio of CD3*ε* was 98.7%, the ratio of CD4 was 97.4%, and the ratio of CD3*ε*+CD4 was 97.6%. The obtained CD4^+^ T lymphocytes could be used for the following experiment.

Additionally, the functions of LPS and PF on the cell viability of splenocytes and CD4^+^ T lymphocytes from MRL/MP mice and MRL/lpr mice were studied. First, we observed the effects of LPS and PF drug concentrations on splenocytes and CD4^+^ T lymphocytes for further exploration. It was found that LPS could dramatically increase the viability of splenocytes and CD4^+^ T lymphocytes at concentrations of 0.001–10 *μ*g/mL (Figure 1(b)). We discovered that 10 *μ*g/mL of LPS had the most apparent effect, as previously reported [29]. Paeoniflorin was one of the active ingredients in *Paeoniae Radix*. When the concentration of PF was 10, 25, or 50 *μ*mol/L, it would not have a toxic inhibitory effect on splenocytes and CD4^+^ T lymphocytes (Figure 1(b)). Subsequently, it was verified whether PF had an effect on the cell viability of splenocytes and CD4^+^ T lymphocytes. As shown in Figure 1(c), PF reduced the cell viability of LPS-induced cells in a concentration-dependent manner. However, after 24 h of drug intervention, the concentration of PF as high as 50 *μ*mol/L showed no significant cytotoxicity to the LPS-treated cells, which indicated that this concentration could be used for further experiments.

### 3.2. PF Inhibits Inflammatory Cytokines Release In Vitro and In Vivo

**Figure 2:**
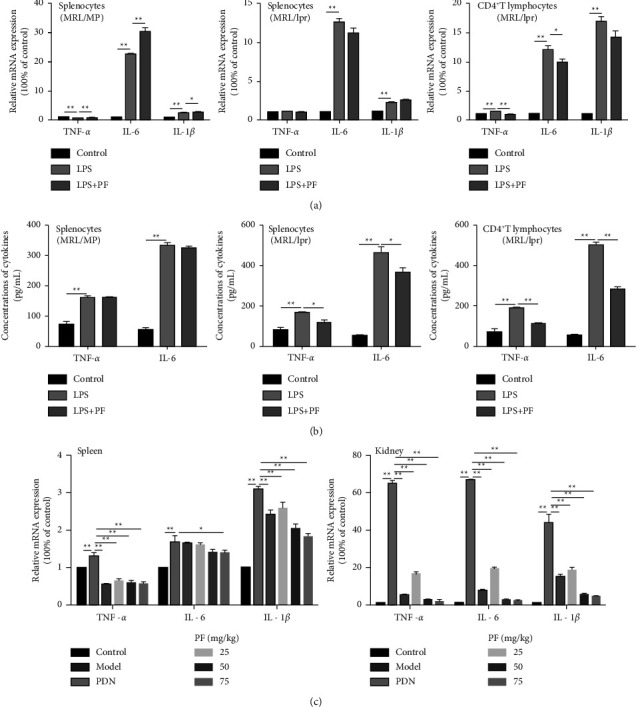
PF inhibits inflammatory cytokines release in vivo and in vitro. (a) TNF-*α*, IL-6, and IL-1*β* mRNA expressions were evaluated by real-time PCR after treating with LPS and LPS in the presence of PF. (b) Levels of TNF-*α* and IL-6 in cell supernatant were measured by ELISA. Splenocytes of MRL/MP, splenocytes of MRL/lpr, and spleen CD4^+^ T lymphocytes of MRL/lpr were treated with 10 *μ*g/mL LPS either alone or with 50 *μ*mol/L PF for 24 h. (c) The mRNA expressions of TNF-*α*, IL-6, and IL-1*β* in MRL/lpr mice. Cells in the control group were treated with vehicle (1640 with 10% FBS). MRL/MP mice treated with distilled water were used as the control group, and MRL/lpr mice treated with distilled water were used as the model group. The results were shown as mean ± S.D (*n* = 3). ^*∗*^ and ^*∗∗*^ indicated significant differences at the levels of *p* < 0.05 and *p* < 0.01.

To determine the role of PF in the expression of inflammatory cytokines in MRL/lpr and MRL/MP mice, the splenocytes and CD4^+^ T lymphocytes were treated with LPS alone or in combination with PF, and the expressions of TNF-*α*, IL-6, and IL-1*β* were detected by Rt-qPCR and ELISA (Figures 2(a) and 2(b)). It was reported that the expressions of TNF-*α* and IL-6 were generally higher in SLE patients [34]. TNF-*α* and IL-1*β* stimulate innate immune response and expand inflammatory cascade by simulating the secretion of other inflammatory cytokines [25,35]. After LPS stimulation, the expressions of TNF-*α*, IL-6, and IL-1*β* in the cells of MRL/lpr mice were up-regulated, especially in CD4^+^ T lymphocytes. This demonstrated that CD4^+^ T lymphocytes might be involved in the pathogenesis of inflammation in MRL/lpr mice. After PF intervention, the inflammatory cytokines in splenocytes and CD4^+^ T lymphocytes of MRL/lpr mice decreased significantly, which was consistent with our previous study of PF on peritoneal macrophages in lupus mice [26]. So, we speculated that PF might inhibit the secretion of inflammatory cytokines in different immune cells of lupus mice. Notably, PF restricted inflammatory cytokines more significantly in CD4^+^ T lymphocytes from MRL/lpr mice. These data illustrated that PF markedly diminished the release of inflammatory cytokine in lupus cells induced by LPS.

The above results were based on in vitro experiments in MRL/lpr mice. To better understand the intervention effect of PF on SLE, we detected inflammatory cytokines mRNA in the spleen and kidney of mice. It was revealed that the expressions of TNF-*α*, IL-6, and IL-1*β* in the spleen and kidney were dramatically increased in the model group (Figure 2(c)). Of note, the TNF-*α*, IL-6, and IL-1*β* mRNA expressions in the PF group and PDN group were significantly decreased. Taken together, PF could suppress the secretion of inflammatory cytokines in MRL/lpr mice.

### 3.3. PF Relieves the Progression of Lupus Symptoms in MRL/lpr Mice

**Figure 3:**
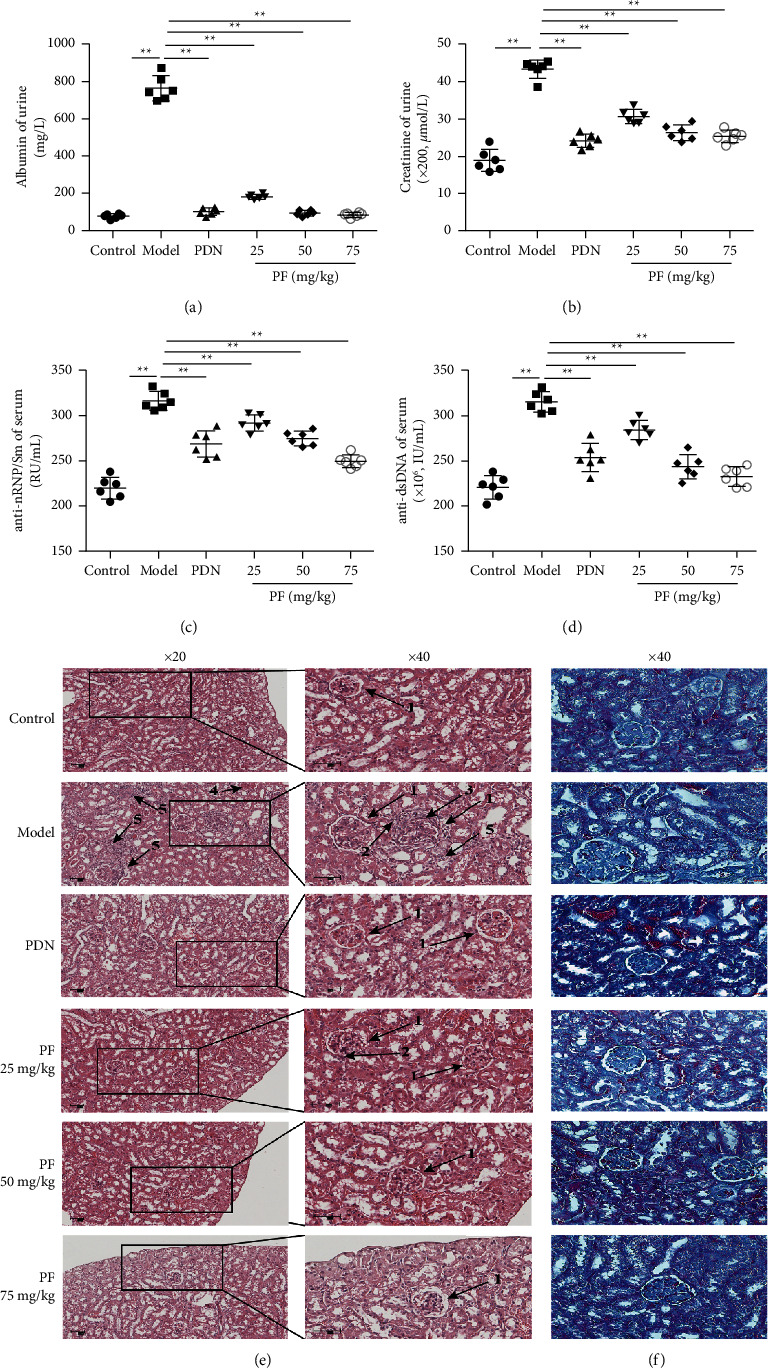
PF relieves the development of lupus symptoms in MRL/lpr mice. Mice were treated with PF (25, 50, and 75 mg/kg) by intragastric administration every day for 8 weeks. Eight weeks later, the urine, serum, and kidneys from mice were harvested and subjected to ELISA and H&E staining. (a, b) Levels of albumin and creatinine in urine were measured by ELISA. (c, d) Levels of anti-nRNP/Sm and anti-dsDNA in serum were measured by ELISA. (e) Representative histopathology images from each group of MRL/lpr mice were shown at ×20 and ×40 magnifications. Scale bar, 50 *μ*m. The arrows indicate the pathology of the kidney. (1) Glomerulus, (2) glomerular mesangial cells, (3) mesangial matrix, (4) renal interstitial congestion, and (5) inflammatory cells. (f) Representative kidney Massion staining of different groups. Scale bar, 50 *μ*m. MRL/MP mice treated with distilled water were used as the control group, and MRL/lpr mice treated with distilled water were used as the model group. The results were shown as mean ± S.D (*n* = 6). ^*∗*^ and ^*∗∗*^ indicated significant differences at the levels of *p* < 0.05 and *p* < 0.01.

Previous experiments have shown that MRL/lpr mice show obvious symptoms of lupus, the most serious of which is kidney disorders [21]. Meanwhile, serious changes in urine protein, urinary creatinine, blood indicators, and kidney function could also be found. To further assess the effect of PF on the improvement of pathological symptoms in MRL/lpr mice, ELISA was used to determine the concentration of urine albumin, urinary creatinine, serum anti-dsDNA, and anti-nRNP/Sm (Figures 3(a)–3(d)). The data demonstrated that urinary albumin and urinary creatinine markedly decreased in MRL/lpr mice after PF intervention (Figures 3(a) and 3(b)). Meanwhile, the anti-nRNP/Sm and anti-dsDNA levels of serum were dramatically reduced in the PF group (Figures 3(c) and 3(d)).

The increased size and swollen state of glomerulus were found in the model group, which lead to severer inflammatory cell infiltration, proteinuria, and glomerular lesions (Figure 3(e)). However, such pathological changes were alleviated by PF intervention, and obvious congestion and massive inflammatory cell infiltration in the renal interstium could not be found. The renal glomerular lesions of the mice in the PDN group were less obvious, while the kidney structure of the control group was relatively complete, with normal glomerular morphology and no prominent pathological changes. Meanwhile, Masson staining showed noticeable glomerular swelling and interstitial fibrosis in the kidney tissue of the model group. After PDN and PF treatment, the above performance was greatly improved (Figure 3(f)). These results elucidated that PF alleviated the progression of lupus symptoms in MRL/lpr mice.

### 3.4. IRAK1 and Its Phosphorylation Are Suppressed by PF in MRL/lpr Mice

**Figure 4:**
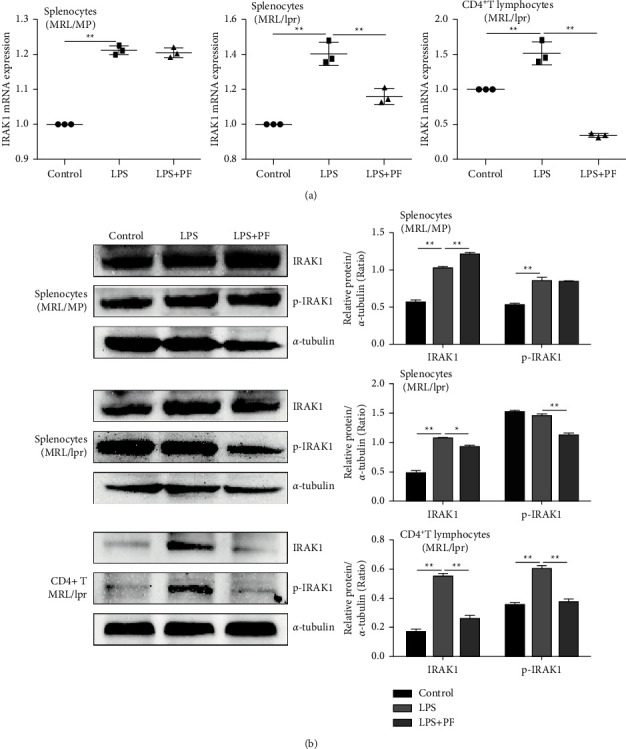
PF suppresses the activation of IRAK1 in vitro. (a) Level of IRAK1 mRNA expression was determined by real-time PCR after splenocytes of MRL/MP, splenocytes of MRL/lpr, and spleen CD4^+^ T lymphocytes of MRL/lpr were treated with 10 *μ*g/mL LPS alone or with 50 *μ*mol/L PF for 24 h. (b) Level of IRAK1 protein expression was determined by Western Blot. The protein bands of IARK1and p-IARK1 and quantitative analysis of protein expression alteration in splenocytes of MRL/MP, splenocytes of MRL/lpr, and spleen CD4^+^ T lymphocytes of MRL/lpr were showed. Cells in the control group were treated with vehicle (1640 with 10% FBS). The results were shown as mean ± S.D (*n* = 3). ^*∗*^ and ^*∗∗*^ indicated significant differences at the levels of *p* < 0.05 and *p* < 0.01.

As a critical regulatory molecule in TLR, IRAK1 is also one of the potential therapeutic targets in SLE. And it is valuable to inhibit the IRAK1 signaling pathway to reduce the inflammatory cascade-mediated tissue damage [27]. In the previous study, we used lentiviral transfection technology to overexpress shRNA with IRAK1 to prove that TCM reduced IRAK1 expression in peritoneal macrophages of MRL/lpr mice [36]. Thus, we evaluated IRAK1 expression levels in vivo and in vitro to further understand its role in the inflammatory pathway through gene and protein detection experiments. As shown in Figure 4(a), LPS could activate IRAK1 mRNA expression in cells of MRL/lpr mice. However, PF intervention only decreased the expression of IRAK1 protein in splenocytes and CD4^+^ T lymphocytes of MRL/lpr mice (Figure 4(b)). This proved that PF had a particular regulatory effect on the expression of IRAK1 in MRL/lpr mice.

**Figure 5:**
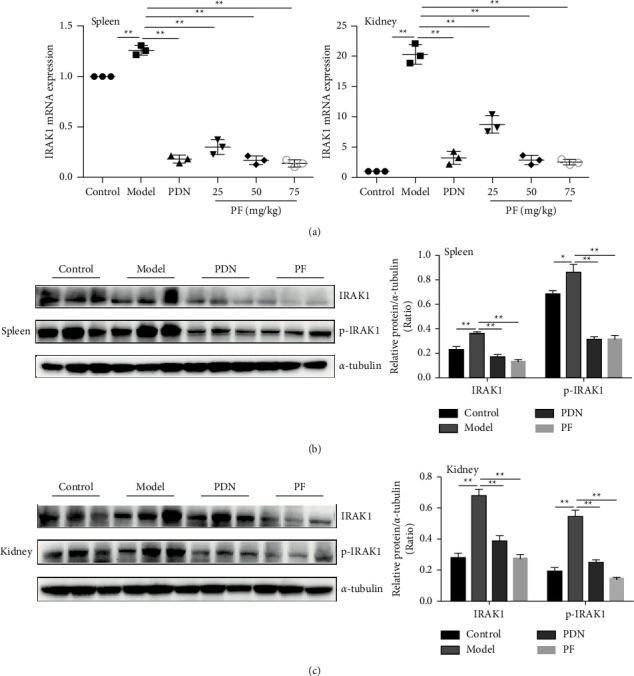
PF inhibits the activation of IRAK1 in vivo. (a) Levels of IRAK1 mRNA in MRL/lpr mice were measured by real-time PCR. (b) The protein bands of IARK1and p-IARK1. (c) Quantitative analysis of protein expression alteration in spleens and kidneys of MRL/lpr. MRL/MP mice treated with distilled water were used as the control group, and MRL/lpr mice treated with distilled water were used as the model group. The therapeutic concentration of PF is 50 mg/kg. The results were shown as mean ± S.D (*n* = 3). ^*∗*^ and ^*∗∗*^ indicated significant differences at the levels of *p* < 0.05 and *p* < 0.01.

To further confirm the therapeutic effect of PF on lupus mice, cDNA and protein were extracted from the spleen and kidney tissues, and the expression of IRAK1 was determined (Figures 5(a)–5(c)). Additionally, we evaluated the phosphorylated expression of IRAK1. As we observed, the expression of IRAK1 and p-IRAK1 in the spleen and kidney was dramatically increased in the model group, indicating that the IRAK1 molecule was activated (Figures 5(b) and 5(c)). Nevertheless, PF and PDN could reduce the expression of IRAK1. These results proved that PF suppressed the activation of IRAK1 molecules in MRL/lpr mice.

### 3.5. PF Down-Regulates IRAK1 Downstream NF-*κ*B Pathway in MRL/Lpr Mice

**Figure 6:**
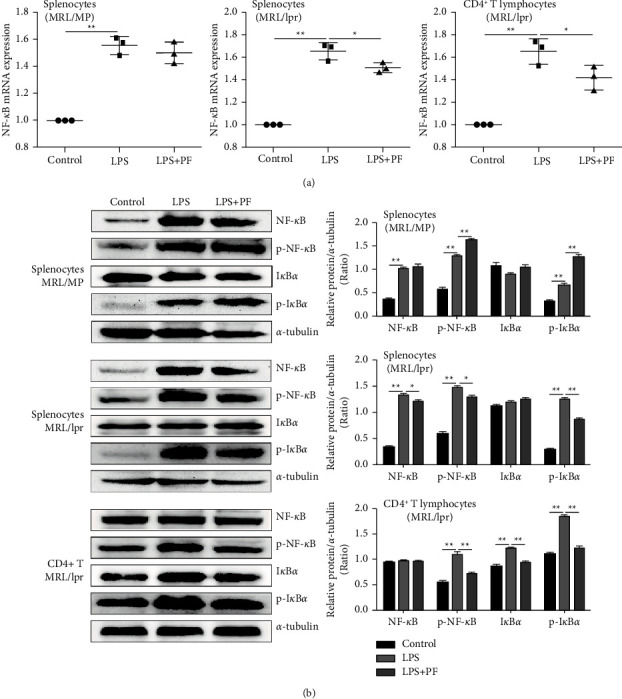
PF suppresses the activation of the downstream NF-*κ*B signaling pathway in vitro. (a) Expressions of NF-*κ*B mRNA in cells were determined by real-time PCR. Splenocytes of MRL/MP, splenocytes of MRL/lpr, and spleen CD4^+^ T lymphocytes of MRL/lpr were treated with 10 *μ*g/mL LPS either alone or with 50 *μ*mol/L PF for 24 h. (b) Levels of IKK*α*/*β*, p-IKK*α*/*β*, I*κ*B*α*, p-I*κ*B*α*, NF-*κ*B, and p-NF-*κ*B protein expression in cells were determined by Western Blot. The protein bands of IKK*α*/*β*, p-IKK*α*/*β*, I*κ*B*α*, p-I*κ*B*α*, NF-*κ*B, and p-NF-*κ*B and quantitative analysis of protein expression alteration in splenocytes of MRL/MP, splenocytes of MRL/lpr, and spleen CD4^+^ T lymphocytes of MRL/lpr were showed. Cells in the control group were treated with vehicle (1640 with 10% FBS). The results were shown as mean ± S.D (*n* = 3). ^*∗*^ and ^*∗∗*^ indicated significant differences at the levels of *p* < 0.05 and *p* < 0.01.

**Figure 7:**
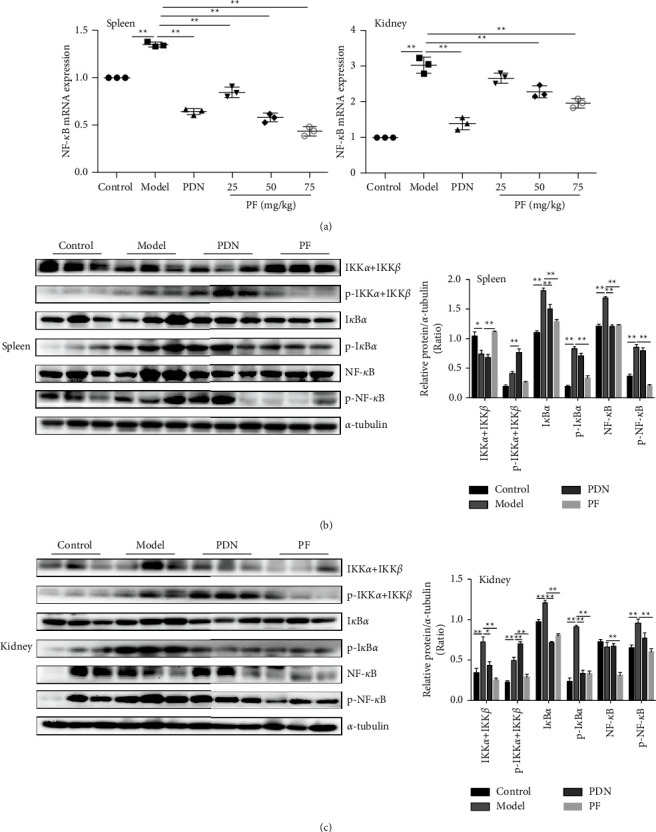
PF inhibits the activation of the downstream NF-*κ*B signaling pathway in vivo. (a) Expressions of NF-*κ*B mRNA in mice were determined by real-time PCR. (b) The protein bands of IKK*α*/*β*, p-IKK*α*/*β*, I*κ*B*α*, p-I*κ*B*α*, NF-*κ*B, and p-NF-*κ*B. (c) Quantitative analysis of protein expression alteration in spleens and kidneys of MRL/lpr were showed. MRL/MP mice treated with distilled water were used as the control group, and MRL/lpr mice treated with distilled water were used as the model group. The therapeutic concentration of PF was 50 mg/kg. The results were shown as mean ± S.D (*n* = 3). ^*∗*^ and ^*∗∗*^ indicated significant differences at the levels of *p* < 0.05 and *p* < 0.01.

NF-*κ*B has been confirmed to have an abnormal active expression in SLE. IRAK1 is a crucial regulator of the NF-*κ*B pathway, so it is speculated that if the upstream IRAK1 molecule is abnormally expressed, the downstream NF-*κ*B signaling pathway can be activated. The expressions of NF-*κ*B in CD4^+^ T lymphocytes of MRL/lpr mice were increased dramatically after LPS stimulation, and also decreased markedly after PF intervention (Figures 6(a) and 6(b)). Nevertheless, this inhibitory effect was less evident in the splenocytes of MRL/MP mice. The results revealed that LPS induction and PF intervention significantly changed the expression of NF-*κ*B in MRL/lpr mouse cells, especially CD4^+^ T lymphocytes. In addition, PF suppressed the expression of NF-*κ*B mRNA and p-NF-*κ*B protein in the spleen and kidney of MRL/lpr mice (Figures 7(a)–7(c)).

LPS would lead to a series of intracellular inflammatory signals which included IKK/I*κ*B/NF-*κ*B activation by binding to TLR4 [37]. The IKK complex can phosphorylate I*κ*B*α*, and then NF-*κ*B protein can be transferred to the nucleus to mediate inflammation. Regarding this pathway, we performed western blotting to observe the expression of IKK*α*/*β* and I*κ*B*α* and their phosphorylation. The results illustrated that the phosphorylation of IKK*α*/*β* in the spleen and kidney was significantly increased in the model group and decreased after intervention with PF (Figures 7(b) and 7(c)). As the phosphorylation of IKK*α*/*β* directly affected the expression of I*κ*B*α* directly, the phosphorylation level of I*κ*B*α* in the spleen and kidney was dramatically reduced in the PF group, consistent with the results of IKK*α*/*β*. The protein data indicated that PF decreased the expression of I*κ*B in the splenocytes and CD4^+^ T lymphocytes of MRL/lpr mice pretreated with LPS, but it was not evident in the splenocytes of MRL/MP mice (Figure 6(b)). Taken together, PF suppressed the activation of the NF-*κ*B inflammatory signaling pathway in MRL/lpr mice.

## 4. Discussion

Our research demonstrated that PF significantly inhibited the activation of the IRAK1 and its downstream NF-*κ*B inflammatory signaling pathway in CD4^+^ T lymphocytes of MRL/lpr mice. These results provide experimental evidence for the relationship between CD4^+^ T lymphocytes and the pathogenesis of SLE inflammation. Notably, we observed that MRL/lpr mice treated with PF exhibited slighter kidney damage and fewer autoantibodies and inflammatory factors. Taken together, these data verified our hypothesis that PF might inhibit the activation of the IRAK1-NF-*κ*B inflammatory signals to treat inflammation in SLE mouse models.

In recent years, new anti-inflammatory drugs have attracted more and more attention due to their effective properties and fewer side effects [38]. Innovative Chinese medicines, such as artemisinin, ligustrazine, and berberine, have become common clinical drugs for their stable quality and strong efficacy. PF had the advantages of a wide range of sources, simple extraction, and pleiotropic effects [39]. Moreover, PF reduced inflammation and tissue damage in autoimmune diseases because it could balance immune cell subpopulations and regulate abnormally activated signal pathways [23,24]. Besides, total glucosides of paeony (TGP) is a drug approved by the China Food and Drug Administration for the treatment of rheumatoid arthritis (RA) and other autoimmune diseases [24,40]. PF, the main active ingredient of TGP, has been reported as a promising drug for autoimmune diseases and inflammatory diseases [23]. Nevertheless, the mechanism of PF on SLE has not yet been determined, and further specific functional studies are still needed. Existing studies proved that PF had a protective effect on liver injury by inhibiting TLR4/MyD88/NF-*κ*B signaling in MRL/lpr mice [41]. Furthermore, JP, a commonly used prescription for the treatment of SLE in Zhejiang Provincial Hospital of TCM, was analyzed by HPLC and LC-MS. Interestingly, we found that PF was the most important active component in JP [36]. Therefore, we tested the possible effective inhibitory effect of PF on the inflammatory response of SLE. Studies have suggested that IRAK1 antagonism can reduce the production of TNF-*α* and IL-6 [42]. However, it was not confirmed whether PF had a similar antagonistic effect on IRAK1. Hence, this study aimed to explore the possible anti-infection-related mechanisms of PF in treating SLE through more in-depth in vivo and in vitro experiments.

At present, MRL/lpr mice are internationally recognized as the most classical animal models of SLE, and their autoimmune disease symptoms are similar to those of SLE patients, including obvious serum autoantibodies and kidney damage [16]. In this study, the kidney pathological sections of the model group showed typical nephritis features such as glomerular cell swelling, inflammatory cell infiltration, mesangial matrix proliferation, and interstitial fibrosis. Meanwhile, the model group showed significantly elevated proteinuria content and higher levels of autoantibodies in the serum, such as anti-dsDNA and anti-nRNP/Sm.

T cells played an essential role in the pathogenesis of SLE and could amplify the autoimmune response when its tolerance was impaired [43]. Published studies demonstrated that overactive autoreactive T cells in SLE produced a large number of autoantibodies to form immune complexes and secreted various inflammatory cytokines, resulting in cell signaling, tissue inflammation, and organ damage [44–46]. As one of the therapeutic targets of SLE, TLR4 is also the primary source of inflammatory cytokines and participates in various inflammatory responses [47,48]. Due to the TLR activation of the adaptor protein MyD88, NF-*κ*B is activated, which leads to the secretion of inflammatory cytokines [49]. LPS is reported to play an important role in the pathogenesis of SLE according to previous experiments. As the ligand of TLR4, LPS is an important component of the outer membrane of Gram-negative bacteria and can induce the expression of inflammatory cytokines in splenocytes in vivo. Indeed, we found that LPS promoted the secretion of inflammatory cytokines in the splenocytes and CD4^+^ T lymphocytes of MRL/lpr mice. In addition, recent research reported that IRAK1 was overexpressed in the PBMC of SLE patients [27]. Dihydroartemisinin (DHA) could inhibit TLR4 expression in splenocytes of MRL/lpr mice by reducing the TLR4-mediated inflammatory signal pathway triggered by LPS [29]. All the above findings confirm our results. After LPS stimulation, the expression of the IRAK1-NF-*κ*B inflammatory signaling pathway in splenocytes and CD4^+^ T lymphocytes of MRL/lpr mice was higher than that of MRL/MP mice. Interestingly, we found that CD4^+^ T lymphocytes had more obvious indicators of inflammation compared with the splenocytes of MRL/lpr mice. Studies have found that CD4^+^ T cells are dysfunctional in SLE [32,33,50], which may explain the active role of CD4^+^ T lymphocytes in the pathogenesis of SLE. As a conclusion, it was speculated that targeting CD4^+^ T lymphocytes might be a new strategy for the treatment of SLE.

SLE is a severe systemic autoimmune disease in which immune complexes are deposited on blood vessels, leading to the recruitment of complement factors and severe inflammation and tissue destruction of the organs such as the spleen and kidney [51–53]. Much evidence indicated that the spleen of MRL/lpr mice showed an enlarged pathological state, but the pathogenesis of SLE splenomegaly was still unknown [54,55]. Although the spleen is not a common target organ of SLE, it plays a vital role in antibody production. However, it is essential to understand the pathogenesis of lupus splenomegaly and the inflammation of the spleen [54]. In the present study, we found that the level of inflammatory factors and the expression of the IRAK1-NF-*κ*B pathway in the spleen of the model group were increased, and the spleen inflammation was alleviated after PF intervention. Almost certainly, kidney involvement is a serious complication of SLE, and its severity is closely related to the prognosis of SLE. More seriously, the replacement of fibrotic tissue and the destruction of normal renal parenchyma could be found [56]. As we found in the model group, the urine protein was markedly increased in the model group, and the pathological section of the kidney showed severe nephritis. However, these symptoms in mice in the PF group were appropriately relieved. Similar to the spleen results, PF could also inhibit the secretion of inflammatory factors and the expression of inflammatory signals in the kidney of MRL/lpr mice. Generally, interventions on the kidney were more obvious than those on the spleen. According to reports, CD4^+^ T cells mediated the induction of autoantibodies to aggravate lupus nephritis [57]. Therefore, we tried to extract immune cells (including T cells, macrophages, etc.) from the kidney to further observe the anti-inflammatory mechanism of PF.

Studies have shown that IRAK1-deficient mice have sufficient immune responses and are less sensitive to systemic autoimmunity [12,58]. Consequently, we hypothesized that the reduction of inflammation in the SLE mouse model might be the result of inhibiting the activity of IRAK1. IRAK1 regulates the expression of inflammatory genes through TLR ligands or IL-1 family members [59]. And further signal transduction leads to transcriptional activators, including NF-*κ*B regulators [60,61]. In this study, a consistent conclusion that the expressions of NF-*κ*B decreased in the spleen and kidney of MRL/lpr mic after PF intervention were reached. Furthermore, the combination of LPS and TLR4 could lead to the activation of the IKK/I*κ*B/NF-*κ*B inflammation pathway [62]. We found that the expressions of IKK*α*/*β* and I*κ*B*α* in MRL/lpr mic were significantly reduced after PF intervention. Notably, clinical studies have elucidated that IRAK1 is overexpressed and can be highly activated in CD4^+^ T cells in SLE patients [63]. Our data support that CD4^+^ T lymphocytes induced by LPS showed a higher level of I*κ*B*α* expression, and its expression was markedly reduced after PF intervention.

## 5. Conclusion

**Figure 8:**
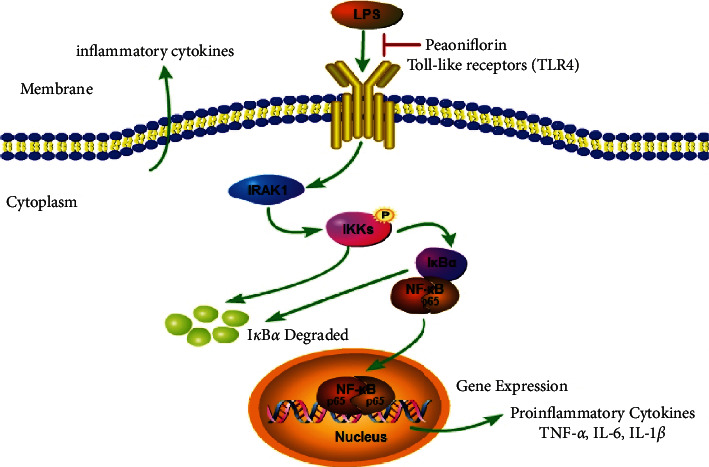
Schematic illustration of the potential protective effect of PF on the progression of SLE. PF ameliorates the inflammatory activity caused by diseases through the IRAK1/IKK*α*/*β*/NF-*κ*B signaling pathway in the process of SLE.

PF could better inhibit the inflammatory activity in CD4^+^ T lymphocytes than that in the splenocytes of MRL/lpr mice induced by LPS. Meanwhile, PF could also alleviate the inflammation in the kidney and spleen of MRL/lpr mice. The anti-inflammatory effect of PF was related to the regulation of IRAK1-NF-*κ*B signaling (Figure 8). Our research showed that PF had an influential role in the anti-inflammatory mechanism of MRL/lpr mice, and inhibiting the IRAK1-NF-*κ*B pathway might be a unique and promising treatment strategy for lupus inflammation.

## Data Availability

The data used to support the findings of this study are available from the corresponding author upon request.
